# Immunomodulatory Effect of Mangiferin in Experimental Animals with Benzo(a)Pyrene-induced Lung Carcinogenesis

**Published:** 2013-06

**Authors:** Peramaiyan Rajendran, Thangavel Jayakumar, Ikuo Nishigaki, Ganapathy Ekambaram, Yutaka Nishigaki, Jayabal Vetriselvi, Dhanapal Sakthisekaran

**Affiliations:** 1 Department of Medical Biochemistry, Dr. ALM PG Institute of Basic Medical Sciences, University of Madras, Taramani Campus, Chennai, India;; 2 NPO-International Laboratory of Biochemistry, 1-166, Uchide, Nakagawa-ku, Nagoya 454-0926, Japan;; 3 Department of Pharmacology and Environmental Toxicology, Dr. ALM PG Institute of Basic Medical Sciences, University of Madras, Taramani Campus, Chennai, India;; 4 Department of Botany and Microbiology, A.V.V.M. Sri Pushpam College, Poondi, Thanjavur, India

**Keywords:** polyaromatic hydrocarbon, Benzo(a)Pyrene, mangiferin, immunomodulation

## Abstract

The immunomodulatory activity of mangiferin was studied in various groups of animals. For this study, adult Swiss albino male mice were treated with benzo(a)pyrene, abbreviated as B(a)P, at 50 mg/kg body weight orally twice a week for 4 weeks; and mangiferin was also given orally (pre- and post-initiation of carcinoma) at 100 mg/kg body weight. Immunocompetence and immune complexes as measured by phagocyte index, avidity index, and soluble immune complex (SIC) levels (*p*<0.001), as well as NBT reduction, were decreased in the B(a)P-treated animals;whereas increased levels of immunocompetence were noted in the mangiferin-treated animals given B(a)P (*p*<0.001, *p*<0.05). The levels of immunoglobulins such as IgG and IgM were decreased considerably (*p*<0.001) in the B(a)P-treated animals compared with their levels in the control animals; whereas the IgA level was increased (*p*<0.001). In the mangiferin-treated experimental animals given B(a)P, the levels of IgG and IgM were significantly (*p*<0.001, *p*<0.05) increased whereas the IgA level was decreased compared with those for the B(a)P-treated mice. Oxidative changes in lymphocytes, neutrophils, and macrophages were also measured. The enhanced lipid peroxidation and decreased catalase and superoxide dismutase activities found in the lymphocytes, polymorphonuclear cells (PMN), and macrophages from B(a)P-treated mice were significantly reduced and increased, respectively, by the mangiferin treatment. This study confirms the immunomodulatory effect of mangiferin and shows an immunoprotective role arbitrated through a reduction in the reactive intermediate-induced oxidative stress in lymphocytes, neutrophils, and macrophages.

## INTRODUCTION

Mangiferin is a naturally occurring xanthone glucoside (2-C-β-d-gluco-pyranosyl-1,3,6,7-tetrahydroxyxanthone). It is found widely distributed in plants such as Anacardiaceae and Gentianaceae families (e.g. *Mangifera indica*, mango), especially in the fruit, leaves, bark and bark root. The pharmacology of mangiferin has gained increased attention in recent years owing to its modulatory actions on oxidative mechanisms in various disorders, and on antitumor (1-6), antiviral (7-9), immunomodulatory (6, 10, 11), and radioprotective (12) activities under different experimental conditions. In our previous study, we found that mangiferin significantly reduced the malonaldehyde (MDA) level, a marker of lipid peroxidation, in different tissues, e.g., lung and liver, under oxidative stress, thus offering protection against B(a)P-induced carcinomas in mice (1-5). The present study was aimed at determining the link between the effects of mangiferin on oxidative stress caused by reactive intermediates specific to the immune system and its protective role against immune dysfunction. B(a)P was used *in vivo* to produce reactive intermediates toxic to the immune system.The ability of mangiferin to reverse the reactive intermediate-induced immunological deficits in antibody production and cellular responses was investigated. The mechanism by which B(a)P induced immunosuppression through oxidative stress on the immune system and the protective role of mangiferin against such stress were demonstrated.

## MATERIALS AND METHODS

### Chemicals

Mangiferin, benzo(a)pyrene, and bovine serum albumin were purchased from Sigma Chemical Company, St. Louis, MO, USA. All other chemicals used were of analytical grade.

### Animals

Male Swiss albino mice (7-8 weeks old) weighing about 23-26 g were purchased from King Institute of Preventive Medicine, Chennai, India. The animals were housed under standard conditions of humidity, temperature (25 ± 2 (C), and light (12 hr light/12 hr darkness). They were fed a standard rat pellet diet and had free access to water. This research work on Swiss albino mice was sanctioned and approved by the Institutional Animal Ethics Committee (IAEC No.02/049/04) University of Madras.

### Experimental design

The animals were divided into 5 groups, each consisting of 6 animals. Group I served as control animals and received corn oil as a vehicle. Group II animals were treated with B(a)P (50 mg/kg body weight, given orally twice a week for 4 consecutive weeks, from the 2^nd^ to 6^th^ week of the 18-week experimental period). Group III animals were treated with mangiferin (100 mg/kg body weight, dissolved in corn oil and given orally) from the 1^st^ week (before start of B(a)P treatment) to the 18^th^ week, twice a week. The B(a)P was administered to these animals simultaneously with the mangiferin from the 2^nd^ week to 6^th^ week for the induction of lung cancer. Group IV animals were post-treated with mangiferin (100 mg/kg body weight, dissolved in corn oil) from the 12^th^ week after the start of B(a)p treatment (which was the same as in group II) up to the end of the experimental period. Group V animals were treated with mangiferin alone (as above) for 18 weeks.

At the end of the experimental period, the animals were fasted overnight and then sacrificed by decapitation. The blood was collected with an anticoagulant and was used for counting immunocompetent cells by the method of John (13), for estimation of immune function by using *Candida albicans* according to Seth and Srinivas (14), and for the nitroblue tetrazolium (NBT) reduction test as described by Gifford and Malawista (15). Coagulated blood was used for the determination of IgG, IgA, and IgM levels according to Tennant *et al*. (16) and Satpathy *et al*. (17) and for that of soluble immune complex levels.

### Analysis of cellular oxidative stress

Lymphocytes and neutrophils from blood were separated by the standard Ficoll paque density-gradient centrifugation method. Briefly, peripheral blood was collected and centrifuged at 500× *g* for 40 min to separate the buffy coat, which was then subjected to Ficoll paque (Pharmacia Biotech) density-gradient centrifugation at 500× *g* for 45 min. The interface containing lymphocytes and the pellet containing neutrophils were separated and washed in PBS. Peritoneal lavage cells were obtained and washed in PBS and suspended in RPMI-1640 medium, after which the macrophages among them were separated by discontinuous Percoll density-gradient centrifugation as per the method of Vray and Plasman (18) with slight modifications. Briefly, isosmotic Percoll was prepared by mixing stock Percoll with 0.9% saline solution at ratios calculated to produce working Percoll solutions with specific gravities of 1.030, 1.040, 1.050, and 1.070 g/ml. The peritoneal lavage cells (1 × 10^6^ cells in 1 ml of RPMI-1640) were added to the top of a centrifuge tube containing 5-ml aliquots of Percoll of these 4 densities and centrifuged at 400× *g* for 20 min at 20°C. Macrophages in the gradient fractions were separated from the remaining cells in the pellet containing the RBCs, granulocytes, and mast cells. The viability of the isolated macrophages was found to be greater than 95% by the trypan blue dye exclusion method. Following washes with PBS, the cells (5 × 10^6^) were finally suspended in 1 ml of PBS, sonicated (twice for 20 s each time) at 80W and centrifuged at 10000× *g* for 10 min at 4°C. The resulting supernatants were collected and immediately analyzed for lipid peroxidation. The protein content was estimated by the method of Lowry *et al*. (19). The samples were also analyzed for the activities of enzyme antioxidants such as superoxide dismutase and catalase, along with quantification of the amount of total protein.

### Superoxide dismutase

Superoxide dismutase (SOD) activity was determined in the lymphocytes, neutrophils, and macrophages according to Madesh and Balasubramanian (20). A colorimetric assay involving the generation of superoxide by pyrogallol autooxidation and the inhibition of superoxide-dependent reduction of the tetrazolium dye 3-(4,5-dimethylthiazol-2-yl) 2,5-diphenyl tetrazolium bromide (MTT) to its formation (21) by SOD was measured at 570 nm. The amount of MTT formazan was calculated using the molar extinction coefficient *E*570 of 17,000 M^−1^ cm^−1^. One unit of SOD was defined as the amount of protein required to inhibit the MTT reduction by 50%.

### Catalase

Catalase activity was measured in the lymphocytes, neutrophils, and macrophages by the method of Maehly and Chance (22). The utilization of hydrogen peroxide (substrate) by catalase in the samples was measured spectrophotometrically as a decrease in optical density at 254 nm. The substrate was used at a final concentration of 20 mM in the assay.

### Lipid peroxidation

Lipid peroxidation in lymphocytes, neutrophils, and macrophages was studied by measuring the levels of malonaldehyde as per Ohkawa *et al*. (23). The results were expressed as nanomoles of MDA formed per milligram cell protein in 30 min.

## RESULTS

**Figure 1:**
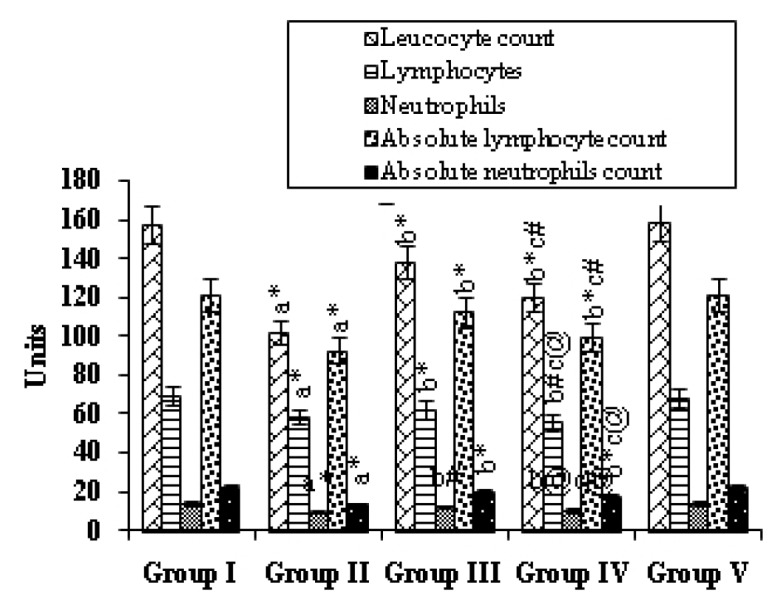
Effect of mangiferin on the hematological counts in control and experimental animals. Each value is expressed as the mean 1 SD for 6 mice in each group. Statistical significance: ^*^ *P*<0.001, ^#^ *P*<0.01, ^@^ *P*<0.05; NS: Not significant; a: as compared with group I; b: as compared with group II; c: as compared with group III. Units: total leucocyte count: no./ mm^3^ × 10^2^; lymphocyte: %; Neutrophils: %; absolute Lymphocyte count: no./mm^3^ × 10^2^; Absolute neutrophil count: no./ mm^3^ × 10^2^.

Fig. 1 presents the effects of mangiferin on the status of immunocompetent cells in the various experimental groups. Group II, i.e., the animals treated with B(a)P, showed a significant *(p*<0.001) decrease in the cell counts when compared with the Group I (control) animals. Mangiferin started pre-initiation (Group-III) caused a significant increase in the counts of leukocytes *(p*<0.001), lymphocytes, and neutrophils, as well as in the absolute numbers of lymphocytes and neutrophils *(p*<0.001) compared with those for Group II. Mangiferin began post-initiation (Group IV) also caused a significant increase in these counts *(p*<0.001, *p*<0.01, *p*<0.05). Treatment with mangiferin alone (Group V animals) resulted in no significant differences in values when compared with those for the control animals.

**Figure 2:**
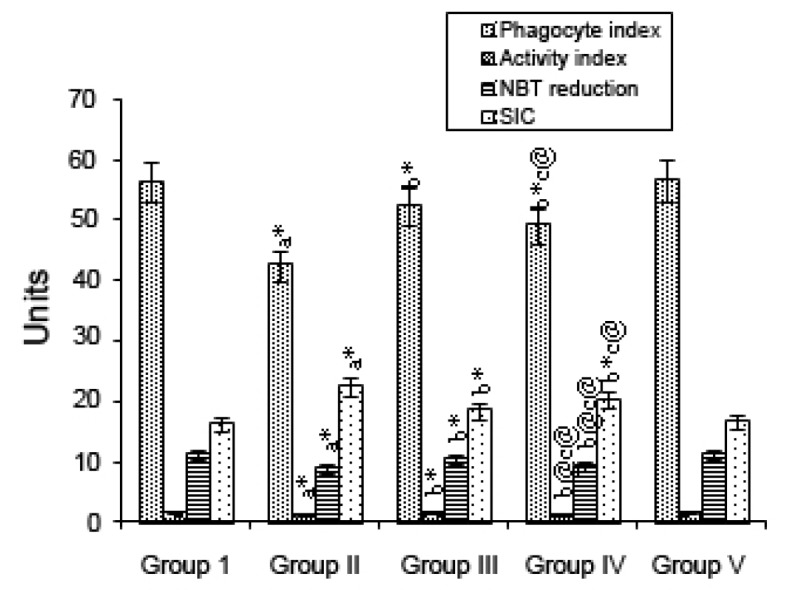
Effect of mangiferin on the phagocyte index, avidity index, NBT reduction, and SIC in control and experimental animals. Each value is expressed as the mean ± SD for 6 mice in each group. Statistical significance: ^*^ *P*<0.001, ^#^ *P*<0.01, ^@^ *P*<0.05; NS: Not significant. a: as compared with group I; b: as compared with group II; c: as compared with group III. Units: phagocytic index, no. of positive cells/100 cells; avidity index, no. of C.albicans /100 positive cells; NBT reduction, no. of positive cells/100 cells; SIC index: PEG index.

The effect of mangiferin on immune complexes as indicated by the phagocyte index, avidity complex, and SIC level and on NBT reduction, in various experimental groups are depicted in Fig. 2. Group II (B(a)P-treated) animals showed a significant *(p*<0.001) decrease in immune complexes when compared with Group I (control) animals. Mangiferin started pre-initiation (Group III) caused a significant *(p*<0.001) increase in the levels of immune complexes compared with the levels for Group II, as did mangiferin treatment started post-initiation (Group IV; *p*<0.001, *p*<0.05). In animals given mangiferin alone (Group V), there were no significant differences in values compared with those for the control animals.

**Figure 3:**
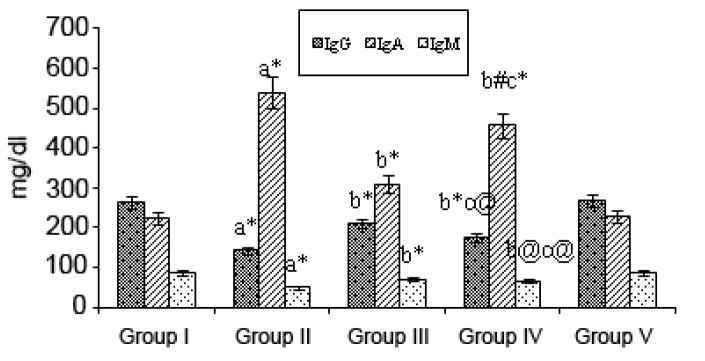
Effect of mangiferin on immunoglobulin levels in the serum of control and experimental animals. Each value is expressed as mean ± SD for 6 mice in each group. Statistical significance: *P<0.001, ^#^ *P*<0.01, ^@^ *P*<0.05; NS: Not significant. a: as compared with group I; b: as compared with group II; c: as compared with group III.

Fig. 3 shows the levels of immunoglobulins IgG, IgA, and IgM in the various experimental groups. IgG and IgM levels were decreased considerably *(p*<0.001) in B(a)P-treated treated Group II animals, along with an increase *(p*<0.001) in the IgA level when compared with the values for the Group I (control) animals. Upon mangiferin treatment started pre-initiation (Group III) the levels of IgG and IgM were significantly *(p*<0.001) increased whereas the IgA level was decreased *(p*<0.01) compared with those for the Group II animals. Mangiferin began post-initiation (Group IV animals) caused considerable alterations in the levels of immunoglobulins *(p*<0.001, *p*<01, *p*<0.05) when compared with those for the Group II cancer-bearing animals. Again, mangiferin given alone had no effects on the normal Ig levels (as seen in Group I).

The B(a)P-treated mice exhibited immunological oxidative stress as revealed by the significant elevation of the malonaldehyde level in the lymphocytes, neutrophils and macrophages, when compared with that level in the control mice. This increase in level was accompanied by a significant decrease in catalase and superoxide dismutase activities. Mangiferin treatment significantly ameliorated the B(a)P-induced oxidative stress as shown by the decrease in the malonaldehyde level accompanied by an increase in the catalase and superoxide dismutase activities in lymphocytes, neutrophils, and macrophages (Table 1).

**Table 1:** Effect of mangiferin on immunological oxidative stress in control and experimental animals

Particulars Group	Lymphocyte			Polymorphonuclear cell			Macrophage		
	SOD	CAT	LPO	SOD	CAT	LPO	SOD	CAT	LPO

**I**	23.43 ± 3.46	56.43 ± 4.23	27.45 ± 1.64	21.32 ± 3.06	10.36 ± 1.13	9.87 ± 1.34	5.65 ± 2.13	3.76 ± 1.05	7.25 ± 0.63
**II**	15.78 ± 2.17	41.93 ± 5.51^a^ *	39.37 ± 4.32^a^ *	11.32 ± 1.05^a^ *>	6.43 ± 0.81^a^ *	21.31 ± 3.81^a^ *	2.23 ± 0.83^a^ *	1.12 ± 0.87^a^ *	13.54 ± 1.32^a^ *
**III**	20.18 ± 1.68	51.27 ± 6.71^b^ *	30.49 ± 3.21^b^ *	19.51 ± 1.17^b^ *	8.89 ± 0.92^b^ *	12.45 ± 2.31^b^ *	4.07 ± 1.65^b^ *	2.54 ± 0.91^b^ *	10.16 ± 2.54^b^ *
**IV**	18.47 ± 2.13	48.35 ± 3.93^b^ * c #	35.71 ± 4.12^b^ # c @	15.78 ± 2.26^b^ * c @	7.16 ± 0.68^b^ @ c ^NS^	16.12 ± 1.72^b^ * c @	3.53 ± 0.43^b^ * c @	1.98 ± 1.10^b^ @ c @	11.42 ± 1.71^b^ @ c @
**V**	24.56 ± 2.20	56.17 ± 4.32	28.31 ± 2.27^b^	20.67 ± 1.50	11.21 ± 0.98	8.98 ± 0.94	5.37 ± 2.43	3.65 ± 1.21	7.43 ± 3.78

Each value is expressed as mean ± SD for six mice in each group. Statistical significance: §: Not significant. Units: SOD; One Unit of SOD the amount of (in μg) of protein required to inhibit the MTT reduction by 50%, CAT; (×10^3^) U (unit for the activity of catalase), LPO; nM MDA /mg protein/ 30 mins. Group I control, group II B(a)P treated, Group III mangiferin pre-initiation, Group IV mangiferin post- initiation, Group V mangiferin alone.

a as compared with group I;

b as compared with group II;

c as compared with group III.

* *P*<0.001,

# *P*<0.01,

@ *P*<0.05.

## DISCUSSION

Polycyclic aromatic hydrocarbons (PAHs) such as B(a)P constitute a major class of widely distributed and persistent environmental contaminants. PAHs are among the most common classes of chemical contaminants found at hazardous waste sites. Their structure consists of 3 or more fused benzene rings that contain only hydrogen and carbon. PAHs are found naturally in crude oil and coal and are formed during the combustion of organic fuels. As a result, they are found in refined oils and fuels, soot, used lubricating oils, crude oil bottoms, and coal products. PAHs produce in most animal species a number of toxic molecules such as carcinogens and have other adverse biochemical consequences, including hepatoxicity, monooxygenase induction, endocrine effects, and immunotoxicity. In general, those compounds containing 1 or more benzene rings fused angularly to a phenanthrene nucleus are considered to be the most physiologically active (24). Many PAHs are known to be potent suppressors of the immune response. The most extensively studied of this class is B(a)P (25). This PAH classically suppresses humoral immune responses but has no effect on cell-mediated or nonspecific immunity, whereas DMBA suppresses humoral, cell-mediated, and nonspecific immunity (25). It is generally thought that the immunosuppressive potential of PAHs is linked to their carcinogenic potency. White *et al*. (26) demonstrated that carcinogenic PAHs suppress the plaque-forming cell (PFC) response in female B6C3F1 mice but that noncarcinogenic PAHs do not; and the rank order of immunotoxic potency nearly matches that of the carcinogenic potency.

B(a)P, a proven carcinogen, is the most prevalent member of the PAH family (27). The first identification of PAH-induced humoral immunosuppression, made by Malmgren *et al*. (28), was followed by a wealth of supportive information regarding immune modulation by PAHs and, in particular, by B(a)P. In our study, a decrease in cell counts, total leukocyte counts as well as absolute neutrophil and lymphocyte counts, were observed in the B(a)P-treated animals. Such decreases may have been due to a decline in the ATP content in the cancerous animals, as most of the activities of the immune cells depend on an adequate cellular energy supply and also due to a poor glycemic condition. In the mangiferin-treated animals given B(a)P the lymphocytes, neutrophils, absolute lymphocytes and absolute neutrophils counts were significantly elevated. The phagocyte and avidity indices and NBT reduction test were found to be significantly decreased in the B(a)P-treated animals when compared with those for the control animals. The killing ability of the neutrophils, as indicated by the NBT reduction, and phagocytic ability of the neutrophils, as indicated by the phagocytic index and the avidity index, were significantly decreased in the B(a)P-treated animals which were further decreased. Serum immune complexes (SIC) serve as an indicator of immune responses due to the presence of either excess antigens or antibodies. In our present study, the B(a)P-treated mice showed markedly increased SIC levels when compared with control animals. This reduction may have been due to the decrease in antibody production in the cancer state. Immunomodulation through natural or synthetic substances may be considered as an alternative for the prevention and cure of neoplastic diseases (29). The rate of immunoglobulin synthesis (IgG, IgM) is reduced in patients with certain neoplastic conditions, indicating diminished humoral immunity and a reduction in immune system response due, in the case of IgG, to an increased non-enzymatic glycosylation of IgG (30). The abnormal features of immunoglobulins in the serum of patients with malignant diseases are well documented (31, 32). Chandy *et al*., (33) reported that the elevated serum IgA levels may be due to the failure of the clearance mechanism in the damaged liver. The immunosuppressive effect of B(a)P is more pronounced in depressed animals, and the effects of B(a)P on the activity of enzymes for energy metabolism differ between normal and depressed mice. It can be hypothesized that simultaneous exposure to adverse social and toxic factors determines a qualitatively new response of the organism (34). The levels of the IgG and IgM immunoglobulins in the mangiferin-treated animals given B(a)P showed a significant increase, whereas the IgA level was decreased.

Further, the possible mechanism by which mangiferin protects against immunological tissue injury may be through the regulation of antioxidant enzyme activities, thus potentiating the cellular antioxidant capacity. However, little is known about the mechanisms including the biochemical changes in the endogenous antioxidant defenses that might be involved in the immune dysfunctions associated with B(a)P immunotoxicity. As well, the immunoprotective role of mangiferin and the mechanism of action for the oxidative immunological injury are yet to be established. The polyphenol mangiferin (MA) has been shown to have important inhibitory effects on macrophage function such as inhibition of phagocytic activity, inhibition of NO production by macrophages stimulated with LPS and IFNγ, and inhibition of extracellular and intracellular production of ROS in macrophages stimulated with phorbol myristate acetate (35, 36).

B(a)P significantly lowered the superoxide dismutase and catalase (CAT) activities in lymphocytes, polymorphonuclear cells (PMN), and macrophages; whereas the administration of mangiferin significantly protected these activities, thus demonstrating that an antioxidant mechanism was involved in its immunoprotective role. The mode of action of B(a)P-induced immunosuppression also involved the stimulation of SOD and CAT, thereby alleviating the lipid peroxidative damage to the lymphocytes, PMN, and macrophages. Mangiferin was earlier reported to scavenge hydroxyl, DPPH, superoxide, and peroxynitrite free radicals as well as lipid peroxides (37, 38). Apart from this, inhibition of lipid peroxidation by mangiferin may have also contributed to some extent to the reduction in the radiation-induced DNA damage from ionizing radiation, which was reported to increase lipid peroxidation (39). This contention is supported by our observation that mangiferin significantly inhibited the generation of H_2_O_2_-induced lipid peroxidation in the B(a)P-treated animals. This reduction in lipid peroxidation by mangiferin may have also contributed to the reduced DNA damage resulting from exposure to γ-radiation, since lipid peroxidation has been reported to damage cellular DNA (40). Mangiferin has also been reported to inhibit radiation-induced depletion of cellular nonprotein sulfhydryl (NPSH) groups. Our present findings can thus possibly explain the immunomodulatory effect of mangiferin, as they revealed an antioxidant effect of mangiferin that ameliorated B(a)P-induced immunological damage. In conclusions, mangiferin showed a potential as one of the immunomodulatory agents. These results suggest that mangiferin exerted a strong immuno stimulant effect.
